# Detection of compound mode of action by computational integration of whole-genome measurements and genetic perturbations

**DOI:** 10.1186/1471-2105-7-51

**Published:** 2006-02-02

**Authors:** Kristofer Hallén, Johan Björkegren, Jesper Tegnér

**Affiliations:** 1 Division of Computational Biology, The Department of Physics and Measurement Technology, Biology and Chemistry, Institute of Technology, Linköping University, S-581 83 Linköping, Sweden; 2 Division of Computational Medicine, Center for Genomics and Bioinformatics, Karolinska Institutet, S-171 77 Stockholm, Sweden; 3 Clinical Gene Networks AB, Karolinska Science Park, Fogdevreten 2B, S-171 77 Stockholm, Sweden

## Abstract

**Background:**

A key problem of drug development is to decide which compounds to evaluate further in expensive clinical trials (Phase I- III). This decision is primarily based on the primary targets and mechanisms of action of the chemical compounds under consideration. Whole-genome expression measurements have shown to be useful for this process but current approaches suffer from requiring either a large number of mutant experiments or a detailed understanding of the regulatory networks.

**Results:**

We have designed an algorithm, CutTree that when applied to whole-genome expression datasets identifies the primary affected genes (PAGs) of a chemical compound by separating them from downstream, indirectly affected genes. Unlike previous methods requiring whole-genome deletion libraries or a complete map of gene network architecture, CutTree identifies PAGs from a limited set of experimental perturbations without requiring any prior information about the underlying pathways. The principle for CutTree is to iteratively filter out PAGs from other recurrently active genes (RAGs) that are not PAGs. The *in silico *validation predicted that CutTree should be able to identify 3–4 out of 5 known PAGs (~70%). In accordance, when we applied CutTree to whole-genome expression profiles from 17 genetic perturbations in the presence of galactose in Yeast, CutTree identified four out of five known primary galactose targets (80%). Using an exhaustive search strategy to detect these PAGs would not have been feasible (>10^12 ^combinations).

**Conclusion:**

In combination with genetic perturbation techniques like short interfering RNA (siRNA) followed by whole-genome expression measurements, CutTree sets the stage for compound target identification in less well-characterized but more disease-relevant mammalian cell systems.

## Background

**Figure 1:**
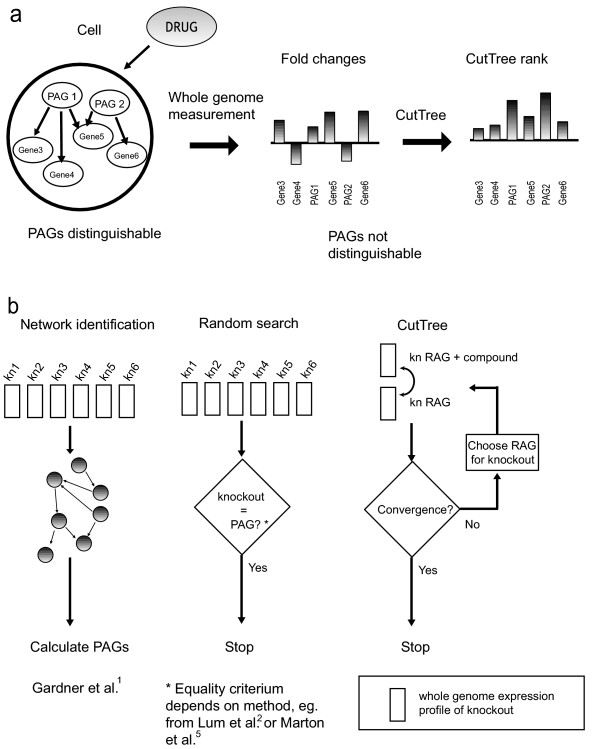
A schematic showing the problem of finding PAGs, existing methods to solve the problem, and the principle of our solution. (a) A compound alters the expression of PAGs in a cell. The change in gene expression then propagates to other genes through the gene network, but genome wide expression analysis only shows a number of expression changes. Thus PAGs cannot be distinguished from their secondary effects from the micro-array data. The CutTree algorithm ranks the genes according to micro-array experiments and identifies the PAGs. (b) To find drug targets, different strategies can be used. With a network identification method (left), the structure and dynamics of the underlying cellular network is identified, and the targets of a compound can be calculated. A random search (middle) can be performed by extending the expression library until the knockout genes coincides with the targets. CutTree (right) infers multiple targets using a small number of experiments.

Identifying the primary targets of chemical compounds determines the selection of compounds suitable for drug development. It is cost effective to improve the accuracy of compound selection since it is a central bottleneck for pharmaceutical companies [1,2]. Furthermore, to reveal the mechanisms of action and toxic side effects of a drug, the primary affected genes (PAGs) must be identified (Fig. 1a). To distinguish the PAGs from indirectly affected genes is a recognized and difficult problem. Currently used methods for PAG identification have limitations. With haploid-insufficiency profiling [3,4], a library of strains with heterozygous deletions is treated with a compound, and those exhibiting growth sensitivity are assumed to be the strains in which the PAGs are deleted. However, the targets must be known deletions and the compound's effect must be reflected by changes in growth. In association analysis, a mutant is assumed to have the PAG deleted if its expression profile is associated with the expression pattern induced by the compound [5]. This strategy is referred to as a random search (Fig. 1b, middle) since the profiles in the libraries are created randomly without choosing specific experiments in the search for PAGs. Gardner et al. succeeded to identify PAGs using an approach (Fig. 1b, left) that however requires a detailed knowledge of the cell network architecture [6], thus denoted as a network identification strategy. Here we design and validate an algorithm, CutTree, which integrates selective experimental perturbations and gene expression measurements, in order to identify PAGs without requiring deletion libraries or knowledge of cellular pathways and network structures (Fig. 1b, right). Validation of CutTree showed it capable of identifying four out of the five primary targets of galactose from 952 differentially expressed genes in Yeast generated from only 17 different gene expression profiles.

## Results

**Figure 2:**
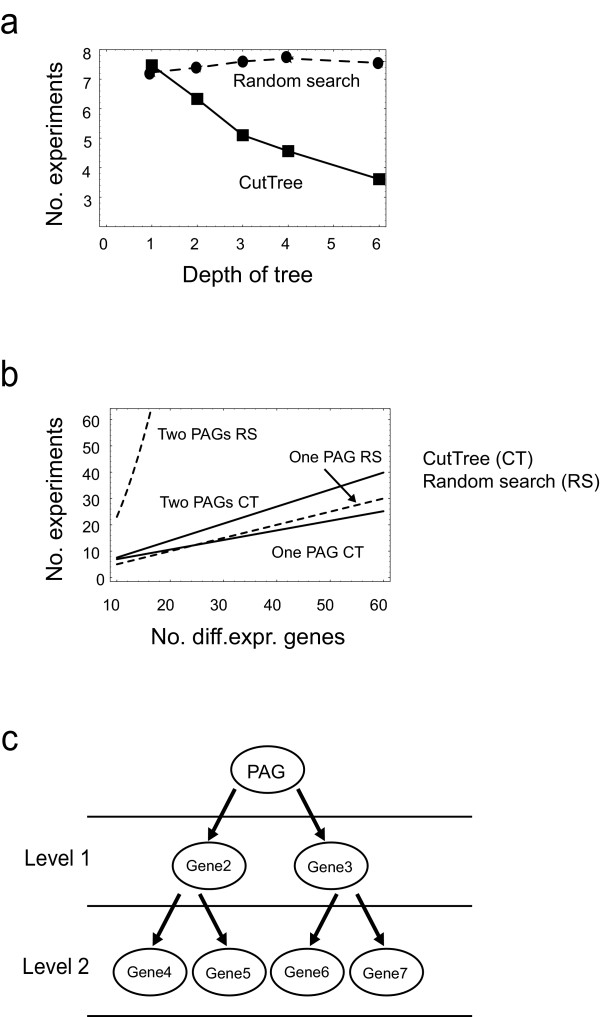
Comparing CutTree with a random search. (a) The structure of the gene network affects the performance of CutTree. A more treelike structure improves its performance. A low depth indicates a shallow tree with many branches; a higher depth indicates a tree with few branches and long paths. Simulation data from 13 *in silico *gene networks with different structures, each being affected by a single PAG. Mean values from 50 experiments are shown (see Methods). (b) *In silico *simulations of the mean number of experiments required to find one PAG among various numbers of differentially expressed genes. For one PAG and a few genes, CutTree performs approximately equal to a random search but performs substantially better as the number of genes increases. For two PAGs, CutTree clearly outperforms a random search. (c) Schematic of gene networks with the PAG at the top.

We have designed and validated CutTree *in silico *and compared CutTree with established methods, primarily the network identification method used by Gardner et al., since the network identification strategy outperforms library based techniques [5]. CutTree defined PAGs with increasing accuracy as the depth of the network tree increased; for example, it defined one PAG in a six-level network in fewer than four experiments (Fig. 2a). A biological network's diameter is proportional to the depth of the tree and is larger than two, regardless of the size of the network [7-9]. It is therefore expected that CutTree should outperform an exhaustive search (based on repeated random perturbations) in identifying PAGs in a biological network. This was confirmed by the *in silico *experiments, demonstrating that the performance of an exhaustive search strategy does not improve with the depth of the tree (Fig. 2a).

**Figure 3:**
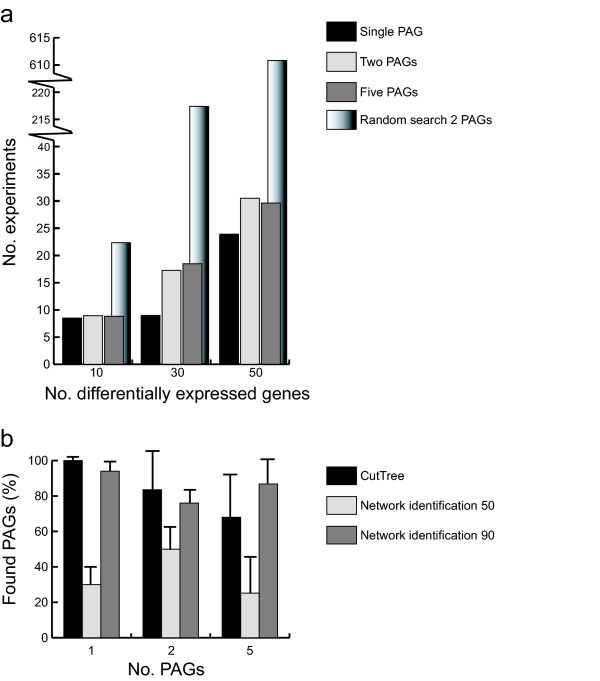
CutTree performance. CutTree outperforms other search methods in gene network simulations, both in the number of experiments needed to find the PAGs and in the number of PAGs found. (a) Average number of experiments needed to find different numbers of PAGs. Average values from 50 *in silico *simulations; the random search bars are the expected values. (b) Comparison between CutTree and the Network identification method. The two Network identification results correspond to 90% and 50% coverage, that is the fraction of identified.

To identify a single PAG the performance was not different between CutTree and an exhaustive search. However, CutTree clearly outperformed an exhaustive search strategy to identify two or five PAGs (Fig. 2b and 3a). The number of genes that are differentially expressed when the compound is applied to the wild type affected the performance of both methods. A large number of differentially expressed genes means a large search space and requires more experiments to find the PAGs (Fig. 2b).

We then compared CutTree with the network identification method developed by Gardner et al [5]. Only when 90% of the network architecture was known, the network identification method could define PAGs with an accuracy which could not be distinguished (P < 2.2 × 10 ^-11^, Student t-test) from that of CutTree (Fig. 3b). However, when 50% of the network connections were known CutTree outperformed the network identification method. In conclusion, the *in silico *experiments clearly demonstrated that CutTree outperforms an exhaustive search strategy (Fig. 1b, middle). The network identification approach (Fig. 1b, left) can only have a similar performance as CutTree when there is a close to perfect understanding (90%) of the underlying biology. Finally, we tested whether these results from CutTree were robust against noise in the data. We found that the noise level had to be larger than 50 % (noise level > 0.5, see Methods for implementation) in order to substantially reduce the performance of CutTree.

**Figure 4:**
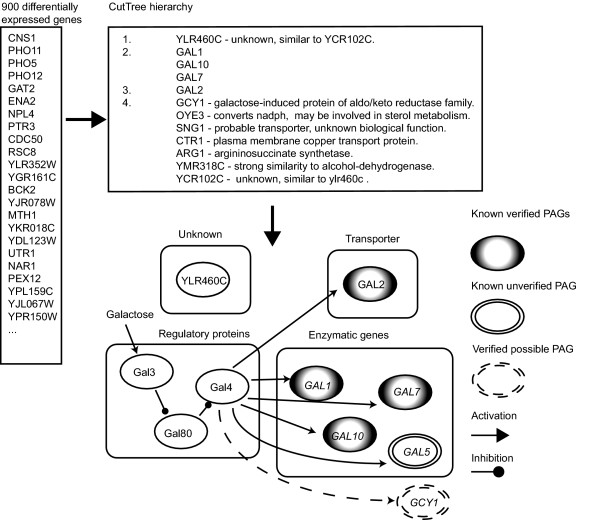
CutTree analysis of data on the Yeast galactose response. CutTree analysis of data on the Yeast galactose response from Ideker *et al*, which showed that in wild-type *S. cerevisiae*, expose to galactose alters the expression of 952 genes. *GAL2 *encodes a transporter that brings galactose into the cell. *GAL1*, *GAL7*, and *GAL10 *encode proteins involved in the conversion of intracellular galactose, and *GAL3*, *GAL4*, and *GAL80 *encode proteins that control the transcription of other GAL genes and each other. When galactose is present, Gal3 binds to Gal80, releasing the repression of *Gal4 *and starting the transcription of enzymatic and transporter genes. Application of CutTree to the data generated a PAG hierarchy. In the top three levels of the hierarchy, four of five genes identified by CutTree are known PAGs.

To experimentally validate CutTree, we applied the algorithm to a whole-genome expression dataset consisting of samples from the eight-gene galactose-response pathway in Yeast (*Saccharomyces cerevisiae*) presented by Ideker et al [10]. This is an appropriate experimental test of the algorithm because GAL1, GAL2, GAL7, and GAL10 are known PAGs for galactose. Galactose induces an ATP-dependent complex consisting of three proteins, Gal3, Gal4 and Gal80 [11]. The binding of Gal4 protein to the complex activates four different genes, GAL1, GAL2, GAL7 and GAL10. Moreover, the GAL5 gene has been suggested to be regulated by the Gal4 protein [12]. Since there are no corresponding measurements of the Gal3-Gal4-Gal80 protein complex in the micro-array experiments [10] we considered these five genes to be PAGs. It has hitherto been unclear whether galactose activation of the Gal4 protein also could activate other genes. Incubating the wild type Yeast with galactose yields more than 900 differentially expressed genes (Fig. 4). To apply CutTree, we transformed the dataset produced by simultaneous application of galactose under 17 different conditions. (6000 transcript measurements under 17 conditions) into 6000 genes under nine conditions (see Methods).

CutTree identified four out of five PAGs in the galactose-response pathway (Fig. 4, left). Note that this excellent performance is almost identical to the predicted accuracy of 70% from the *in silico *experiments (Fig. 3b). Given the large number of possible combinations of five PAGs in a set of 952 differentially expressed genes (10^12^), an exhaustive experimental library search would not have been feasible. Interestingly, not all of the five PAGs are differentially expressed when galactose is applied demonstrating that PAGs could not be identified from inspection of the amount of fold change. In particular, the deleted genes are not as a rule, classified as PAGs by CutTree. For example, knockouts of the GAL3, GAL4, and GAL80 genes, are correctly not labeled as PAGs by CutTree. Thus, neither the fold change nor the knockouts are sufficient to reveal the identity of the PAGs (Fig. 1a). Note that CutTree identified YLR460C, a previously unidentified PAG for galactose but did not detect GAL5 which have been suggested to be a PAG. This could reflect more complex regulation of the PAGs by Gal4, such as an unknown intermediate step between Gal4 and GAL5, possible involving Ylr460c. GAL5 has in contrast to GAL1, GAL2, GAL7, GAL10, not been verified as specifically regulated by Gal4[13]. Furthermore YLR460C is a stress-response gene [14] suggesting that under conditions of stress Gal4 could also regulate this gene. GCY1 (ranked 4) might also be partly controlled by Gal4 [15,16] and could therefore be a PAG. These suggestions are amenable for further experimental validation.

## Discussion

We have designed an algorithm, CutTree, which identifies the mechanisms of action of chemical compounds. CutTree could speed up the drug development pipeline, alleviating the current bottleneck in which the mechanism of action and side effects of current drug candidates are as a rule uncharacterized [2]. Our strategy was to first validate the algorithm using *in silico *experiments and subsequently to test CutTree on public Yeast data. The rational behind our *in silico *evaluation is that for chemical compounds there is yet no clear procedure on how to directly infer mechanism of action from experimental data. It is therefore essential to independently validate and benchmark any algorithm, including CutTree, which specifies an experimental design and data-analysis. Consequently, the *in silico *data is particularly useful since the correct mechanism of action is inherently known by construction of the experiment. Hence, the *in silico *evaluation ensures a proper estimation of the expected success rate and data requirement for inference of mechanism of action before applying the technique in the analysis and design of biological experiments. Our *in silico *analysis demonstrated that CutTree can identify PAGs better than a random search method (Fig. 2a), in particular in the case of several PAGs (Fig. 3a). Furthermore, our analysis predicted a 70% success rate when there are five unknown PAGs (Fig. 3b).

Interestingly, this prediction came in close agreement with the experimental validation where CutTree identified four out of five (80%) of the primary targets of galactose in Yeast using data from 952 differentially expressed genes generated from 17 different perturbations. We may ask how many of the suggested candidates in the list generated by CutTree we would have to consider in order to identify those five PAGs? The estimated 70 % success rate from the *in silico *estimation informs us that if we consider the top 8 candidates, then we can expect 5.6 true PAGs. This procedure would ensure 100 % (5/5) recall, 62.5 % (5/8) precision, thereby a false positive rate of 37.5 % (3/8). Since there is always a trade-off between precision and recall which depends on the priorities of the experimenter there is also a rationale for choosing the top five candidates from the CutTree list with an estimated 3.5 true PAGs, hence a recall of 60 % (3/5) and 60 % (3/5) precision. Now, as the validation using the experimental data demonstrates, choosing the top five candidates, gives 80 % (4/5) and 80 % (4/5) precision. The efficient performance of the CutTree algorithm relies on the fact that CutTree utilizes a simultaneous application of the chemical compound and a genetic perturbation such as a knockout. Therefore, CutTree does not depend on the availability of a priori knowledge, which are currently available only for Yeast (e.g. [17]). Thus, CutTree is complementary to other methods [5] which require large knockout libraries. Even though CutTree does not require prior knowledge, information about putative pathways in which the compound could target facilitates the choice of the suggested initial genetic perturbation in the CutTree algorithm.

A complementary approach to CutTree is to first identify the underlying regulatory network and from the interaction map calculate the PAGs (Fig. 1b). Such a network strategy has a similar performance as CutTree provided that close to 90 % of the interactions in the network have been identified (Figure 2c). In Gardner et al. a network strategy was designed and experimentally tested on E-coli for a previously well characterized small network of nine genes constituting the SOS pathway. However, the applicability of such a network-based approach has hitherto been hampered by the difficulty to identify regulatory networks even in simpler organisms including E-coli and Yeast [7,15]. To perform an estimate of the usefulness of a network based strategy depends on to what extent regulatory networks have been characterized as of today. Clearly, any estimation of our current understanding heavily depends on the individual researcher's assumptions. For example, given that less than 60 % [18] of the open reading frames (ORFs) have been characterized, how large fraction of the Yeast network has thereby been identified? One scenario is that we have complete knowledge of all the interactions between those characterized open reading frames (ORF). From this follows that 64 % of the interactions are unknown, since the interactions between the ORFs only account for 36 % of the total number of interactions. This can be interpreted as an optimistic estimate since most likely there are interactions between ORF that have not yet been characterized. For example, we would only know 18 % of the Yeast network if 50 % of the interactions between the ORFs were known. On the other hand, the estimates used are slightly biased on the negative side since we may have correlation-based information on the interactions between characterized ORFs and uncharacterized ORFs as well as mutual interaction between uncharacterized ORFs. Taking all these approximations together, we believe it is reasonable to argue that no more than about 50 % of all the interactions in Yeast are known. Considering the fact that the Yeast regulatory network probably is the best characterized regulatory network as of today, CutTree should serve as a useful tool for detection of PAGs in Yeast and other organisms, let alone mammalian cells, in the nearest foreseeable future. However, in the last couple of years there has been a rapid progress on algorithms and their application to experimental data for identifying cellular networks, mainly targeting E-coli and Yeast [7,19-22].

Indeed, a recent study [23] has demonstrated the success of a slightly modified network-based approach, here denoted as a mode-of-action by network identification (MNI), where the regulatory interactions in Yeast are estimated from a large knockout compendium. The MNI algorithm correctly enrich for known targets and associated pathways in the majority of compounds that were examined. However, there are a number of differences between CutTree and MNI suggesting different domains of applications for the two algorithms depending on the amount of available prior biological knowledge; (i) The MNI requires large libraries of expression profiles from treated cells in order to train the network model. For Yeast MNI used 515 different expression profiles which were available. (ii) In addition, the MNI requires a large amount of prior knowledge. The MNI network model is based on principal component regression, thus reducing the network model of genes into a network model of metagenes since 515 expression profiles are not sufficient to identify a network model of genes. Note that number of ORFs is more than 10 times larger than the number of gene expression profiles. The PAGs, which were calculated from the compound action on the metagenes, were projected back to the genes. This step is essentially underdetermined and the MNI approach therefore requires a library of pathways and relevant GO classification in order to interpret the ranking of the metagenes. A preliminary analysis showed that the MNI algorithm could not detect any of the galactose PAGs in the top 50 putative PAGs derived from the Yeast gene expression library. The additional GO classification method used in [23] did not produce any significant pathways.

In contrast, CutTree does not require a library of expression profiles or a well-annotated system with pathways. Hence, CutTree is adapted to precisely characterize the mechanisms of action of drugs and chemical compounds in systems where less prior knowledge is available but the disease relevance might be larger than compared to better described systems such as E-coli and Yeast. Furthermore, CutTree provides the experimentalist with a novel experimental design protocol for how to perform a sequential and simultaneous application of a compound and a genetic perturbation, such as a knockout, in order to obtain the most useful information.

## Conclusion

CutTree is complementary to the network identification strategy, including the MNI algorithm. CutTree therefore sets the stage for compound target identification in less well-characterized but more disease-relevant mammalian systems where novel genetic perturbation technologies such as sRNAi are making rapid progress. Further biological experiments will tell whether CutTree is broadly applicable for drug evaluation using prokaryotic and eukaryotic cell lines and, more importantly, whether CutTree proves to be useful in the analysis of compound mode of action in mammalian cells.

## Methods

The underlying idea of the CutTree algorithm is to iteratively define primary affected genes (PAGs) by identifying recurrently active genes (RAGs) – genes whose expression changes most frequently over several whole-genome expression measurements. CutTree provides an experimental design in which the algorithm specifies which genes should be perturbed in the next round of experiments, for a given set of micro-array measurements. In addition, CutTree analyzes the data and produces a candidate list of PAGs. Based on this, CutTree either halts or suggests another genetic perturbation. Importantly, the chemical compound introduces an unknown perturbation to the system and the additional simultaneous well defined genetic perturbation (knockout/siRNA) performed by the experimenter as suggested by CutTree, makes it possible to distinguish the effect of the compound after only a small number of experiments. This is different from current approaches to the mode-of-action problem, where only one genetic perturbation (knockout/siRNA) is used in the absence of the compound (e.g. as in datasets like Hughes et al.) The efficient performance of CutTree depends on the simultaneous application of the compound with a selective genetic perturbation, such as a knockout or an siRNA perturbation [17,24]. In each iteration we allow the knockout/siRNA perturbation target the top RAG. A termination criterion defines and ranks the top candidate PAGs (Fig. 1b, right). This strategy assumes that PAGs are the same genes independently of the second perturbation and that PAGs are at the highest level in the network hierarchy, since the compound constitutes an external perturbation of the root of the tree (Fig. 2c). CutTree iteratively calculates the probability that a gene is a PAG, based on the information obtained by perturbing genome-wide expression. A well-defined stop criterion halts the iteration. We use a Dirichlet distribution with hyper-parameters α_1 _and α_0 _to represent the probability that each gene is a PAG. This is a natural formulation as we have taken a probabilistic approach. The parameters encode a frequency formulation since they are based on the number of times we observe a PAG or nonPAG. The expected value of the distribution follows

α1α1+α0.
 MathType@MTEF@5@5@+=feaafiart1ev1aaatCvAUfKttLearuWrP9MDH5MBPbIqV92AaeXatLxBI9gBaebbnrfifHhDYfgasaacH8akY=wiFfYdH8Gipec8Eeeu0xXdbba9frFj0=OqFfea0dXdd9vqai=hGuQ8kuc9pgc9s8qqaq=dirpe0xb9q8qiLsFr0=vr0=vr0dc8meaabaqaciaacaGaaeqabaqabeGadaaakeaadaWcaaqaaGGaaiab=f7aHnaaBaaaleaaieaacqGFXaqmaeqaaaGcbaGae8xSde2aaSbaaSqaaiab+fdaXaqabaGccqGHRaWkcqWFXoqydaWgaaWcbaGae4hmaadabeaaaaGccqGGUaGlaaa@36B5@

### The CutTree algorithm

1. Choose *n*, the number of genes to use in the stop criterion.

Choose *m*, the number of times the stop criterion shall remain unchanged.

STOP = 0.

For each gene *i*

α_1i _= α_0i _= 1 (uniform distribution).

2. If we have *a priori *knowledge that a gene is likely to be/not be a PAG.

Update α_1 _and α_0 _for all genes accordingly. Here we use 1. Another way would be to set α_1i _= number of times *i *observed as a PAG/number of times *i *observed as a non-PAG.

If gene *i *is likely to be a PAG, increase α_1i _by 1.

If gene *i *is not likely to be a PAG, increase α_0i _by 1.

3. While STOP = 0:

Calculate the expectation values (peaks) of the Dirichlet distributions:

peaki=α1iα1i+α0i.
 MathType@MTEF@5@5@+=feaafiart1ev1aaatCvAUfKttLearuWrP9MDH5MBPbIqV92AaeXatLxBI9gBaebbnrfifHhDYfgasaacH8akY=wiFfYdH8Gipec8Eeeu0xXdbba9frFj0=OqFfea0dXdd9vqai=hGuQ8kuc9pgc9s8qqaq=dirpe0xb9q8qiLsFr0=vr0=vr0dc8meaabaqaciaacaGaaeqabaqabeGadaaakeaacqWGWbaCcqWGLbqzcqWGHbqycqWGRbWAdaWgaaWcbaGaemyAaKgabeaakiabg2da9maalaaabaaccaGae8xSde2aaSbaaSqaaGqaaiab+fdaXmXvP5wqSXMqHnxAJn0BKvguHDwzZbqegyvzYrwyUfgaiqGacaqFPbaabeaaaOqaaiab=f7aHnaaBaaaleaacqGFXaqmcaqFPbaabeaakiabgUcaRiab=f7aHnaaBaaaleaacqGFWaamcaqFPbaabeaaaaGccqGGUaGlaaa@4BF7@

Choose the gene with the largest peak value as the knockout. If multiples genes have the largest peak value, choose one at random as the target for the next knockout.

Perform genome-wide expression profiling to compare the knockout with the knockout in the presence of the compound.

Calculate expression changes.

For each gene *i:*

If expression changed, increase α_1i _by 1.

If expression did not change, increase α_0i _by 1.

Rank genes according to their peaks.

If the *n *highest genes = the *n *highest genes from the last *m *iterations

STOP = 1.

4. End

Note that CutTree does not specify how to perform the differential gene expression analysis since this is general problem within micro-array statistics. Parameters in the algorithm: In the *in silico *experiments, we used *n *= 6 and *m *= 4. PAGs are considered to be identified if they are among the top 10 most significant genes after the algorithm halted. This increases the number of false positives but also makes it more likely that we find the PAGs. Thus, we prefer to select for false positives instead of removing PAGs from the candidate list, increasing the false negative rate. These parameters are flexible depending on the behavior of the stop criterion and the approximate number of expected PAGs, which in turn depends on the particular experimental application.

### Simulated random gene networks

The Mathematica 5.0 (Wolfram Research) package was used for all simulations. The simulated networks were modeled as linear networks. For a network with n nodes we describe x_*i *_as the concentration of RNA from gene *i *and the dynamics follows

dxidt=∑j=1nwijxj,
 MathType@MTEF@5@5@+=feaafiart1ev1aaatCvAUfKttLearuWrP9MDH5MBPbIqV92AaeXatLxBI9gBaebbnrfifHhDYfgasaacH8akY=wiFfYdH8Gipec8Eeeu0xXdbba9frFj0=OqFfea0dXdd9vqai=hGuQ8kuc9pgc9s8qqaq=dirpe0xb9q8qiLsFr0=vr0=vr0dc8meaabaqaciaacaGaaeqabaqabeGadaaakeaadaWcaaqaaiabdsgaKjabdIha4naaBaaaleaacqWGPbqAaeqaaaGcbaGaemizaqMaemiDaqhaaiabg2da9maaqahabaGaem4DaC3aaSbaaSqaaiabdMgaPjabdQgaQbqabaGccqWG4baEdaWgaaWcbaGaemOAaOgabeaaaeaacqWGQbGAcqGH9aqpcqaIXaqmaeaacqWGUbGBa0GaeyyeIuoakiabcYcaSaaa@441F@

where w_ij _are the weights indicating how much gene j affects gene i. This gives an equation system with the concentration vector **x **of length n, the n × n weight matrix A and a vector **b **of length n containing the perturbations, the elements of **b **different from 0 represent the targets of the compound:

dxdt=Ax+b.
 MathType@MTEF@5@5@+=feaafiart1ev1aaatCvAUfKttLearuWrP9MDH5MBPbIqV92AaeXatLxBI9gBaebbnrfifHhDYfgasaacH8akY=wiFfYdH8Gipec8Eeeu0xXdbba9frFj0=OqFfea0dXdd9vqai=hGuQ8kuc9pgc9s8qqaq=dirpe0xb9q8qiLsFr0=vr0=vr0dc8meaabaqaciaacaGaaeqabaqabeGadaaakeaadaWcaaqaaiabdsgaKjabdIha4bqaaiabdsgaKjabdsha0baacqGH9aqpcqWGbbqqcqWG4baEcqGHRaWkcqWGIbGycqGGUaGlaaa@38E5@

Initial values for the simulations were set randomly, and the simulation proceeded until a steady state was reached. When a gene was knocked out, the gene was kept in the matrix but all the connections to that gene were removed and the expression set to zero.

As a rule, the network structure was generated randomly, thus allowing for loops and other network motifs. The out-degree of the network connectivity followed a power law [25]: the probability that a randomly chosen node from the network had *d *outgoing interactions is *P*(*d*) = *d*^-2.3^. In the simulations, 200 nodes (n) were used in the networks, and the node weights (w_ij_) varied between -3 and 3 with a flat distribution. All simulation data are mean values from at least 50 experiments. In evaluating the effects of different network structures (Fig X), small networks (13 genes and one PAG) were created. The structures varied from the PAG being connected to all other genes (shallow tree with many branches and short paths) to the PAG being connected to a few genes (deeper tree with fewer branches and longer paths). To evaluate the effects of noise, we used an additive noise model such as noisy_x_i _= x_i _+ x_i _* noise level, where 0<noise level<1.

In the network identification method (Fig. 1b, left) of Gardner *et al*. the identified network can be represented by a connectivity matrix A. PAGs are found by applying the perturbation to the network (unknown vector **p) **and measuring gene expression (measured vector **x)**. We have the equation system

*x *= *Ap*,

which can be solved.

For comparisons with the Network identification method, we generated random linear networks and performed the simulations as described above. The network was then mutated by randomly removing nodes and inserting random false nodes to reach certain coverage of the original network. The PAGs were then calculated with the Gardner method using the matrix from the mutated network.

### Training data calculations

The data from Ideker *et al*. were recalculated to non-logarithmic values. They are also relative to the reference wild-type + galactose (wt + gal) of the form:

GAL1+galwt+gal=yGAL1−galwt+gal=x.
 MathType@MTEF@5@5@+=feaafiart1ev1aaatCvAUfKttLearuWrP9MDH5MBPbIqV92AaeXatLxBI9gBaebbnrfifHhDYfgasaacH8akY=wiFfYdH8Gipec8Eeeu0xXdbba9frFj0=OqFfea0dXdd9vqai=hGuQ8kuc9pgc9s8qqaq=dirpe0xb9q8qiLsFr0=vr0=vr0dc8meaabaqaciaacaGaaeqabaqabeGadaaakqaabeqaamaalaaabaGaem4raCKaemyqaeKaemitaWKaeGymaeJaey4kaSIaem4zaCMaemyyaeMaemiBaWgabaGaem4DaCNaemiDaqNaey4kaSIaem4zaCMaemyyaeMaemiBaWgaaiabg2da9iabdMha5bqaamaalaaabaGaem4raCKaemyqaeKaemitaWKaeGymaeJaeyOeI0Iaem4zaCMaemyyaeMaemiBaWgabaGaem4DaCNaemiDaqNaey4kaSIaem4zaCMaemyyaeMaemiBaWgaaiabg2da9iabdIha4jabc6caUaaaaa@548C@

The data were rearranged to fit the CutTree algorithm:

GAL1+galGAL1−gal=yx
 MathType@MTEF@5@5@+=feaafiart1ev1aaatCvAUfKttLearuWrP9MDH5MBPbIqV92AaeXatLxBI9gBaebbnrfifHhDYfgasaacH8akY=wiFfYdH8Gipec8Eeeu0xXdbba9frFj0=OqFfea0dXdd9vqai=hGuQ8kuc9pgc9s8qqaq=dirpe0xb9q8qiLsFr0=vr0=vr0dc8meaabaqaciaacaGaaeqabaqabeGadaaakeaadaWcaaqaaiabdEeahjabdgeabjabdYeamjabigdaXiabgUcaRiabdEgaNjabdggaHjabdYgaSbqaaiabdEeahjabdgeabjabdYeamjabigdaXiabgkHiTiabdEgaNjabdggaHjabdYgaSbaacqGH9aqpdaWcaaqaaiabdMha5bqaaiabdIha4baaaaa@4301@

A fold change of two was used as cutoff to classify a gene as differentially expressed or not. This was the only dataset we could identify that contained a dual application of a compound (galactose) and a genetic perturbation (knockout).

## Authors' contributions

KH carried out the *in silico *implementations of CutTree and the validation of CutTree. JB participated in the analysis of the results and draft of the manuscript. JT conceived of the study and participated in its design and the draft of the manuscript. All authors read and approved the final manuscript.
